# F7 promotes osteoclast precursor migration and osteoclastic bone resorption

**DOI:** 10.1515/med-2026-1543

**Published:** 2026-11-06

**Authors:** Huaizhi Zhang, Xu Chen, Jianhui Dai, Haibin Lin

**Affiliations:** Department of Minimally Invasive Spinal Surgery, The Affiliated Hospital of Putian University, Putian, Fujian, China

**Keywords:** coagulation factor VII, osteoclast, migration, bone resorption, osteoporosis

## Abstract

**Objectives:**

Blood coagulation factor VII (FVII, F7) acts as a crucial cytokine that promotes cell migration, and its function is exerted through binding to tissue factor (TF). Migration of osteoclast precursors (OCPs) is essential for osteoclast recruitment, fusion and differentiation. Our study aimed to explore the role of F7 in OCP migration and osteoclastogenesis.

**Methods:**

We first introduced varying concentrations of F7 to observe the migration and differentiation capacities of OCPs. Subsequently, the role of TF in F7-regulated OCP parameters was examined through TF lentiviral silencing. Finally, the effects of F7 on bone parameters, OCP adhesion and osteoclastogenesis in ovariectomized mice were investigated using F7-silenced adeno-associated viruses (AAVs).

**Results:**

The results showed that F7 promoted OCP migration and osteoclastogenesis in a concentration-dependent manner. The enhancing effects of F7 on OCP migration and osteoclastogenesis were blocked by TF silencing. Furthermore, F7 silencing ameliorated bone loss in ovariectomized mice, accompanied by reduced OCP adhesion and impaired osteoclastogenesis within trabecular bone tissue.

**Conclusions:**

Collectively, F7 enhances osteoclastic bone resorption by promoting OCP migration.

## Introduction

Coagulation factor VII (F7), an initiator of the coagulation cascade, can form a F7-TF complex by binding to tissue factor (TF, CD142) to activate downstream signaling pathways. This complex has been confirmed as a key molecular mechanism regulating the migration of various cell types via activating protease-activated receptors. TF-F7-biased signaling is the driving factor of inflammatory cell lung infiltration upon viral nucleic acid response [1]. TF-F7 complex-upregulated Integrin α1 promotes cervical cancer cell migration through PAR2-dependent MEK1/2 activation [2]. Similar reports have also appeared in other studies [3], [4], [5]. These findings imply that F7 may play a role in cell recruitment during bone remodeling.

Migration of osteoclast precursors (OCPs) is a prerequisite for osteoclast recruitment, fusion and final differentiation, directly affecting osteoclastic bone resorption. Abnormal OCP migration is closely associated with bone metabolic diseases such as osteoporosis [6], 7]. In the ovariectomized (OVX) mouse model, enhanced OCP migration leads to excessive osteoclast activation, which is one of the core pathological mechanisms of postmenopausal bone loss [8], 9]. However, whether F7 is involved in regulating OCP migration and osteoclastogenesis remains unclear. Overall, elucidating the regulatory mechanisms of OCP migration is critical for the treatment of osteoporosis. We hypothesize that F7 enhances osteoclastogenesis and bone loss by enhancing OCP migration. The present study aims to address whether F7 regulates OCP migration and subsequent osteoclast formation to affect bone metabolism; this is based on a series of *in vitro* and *in vivo* experiments.

## Materials and methods

### Isolation and induction of osteoclast precursors (OCPs)

OCPs were isolated from bone marrow originated from 4∼6-week-old C57BL/6J mouse tibiae as follows: bone marrow cells were incubated in alfa-MEM (12561–056, Gibco) with 10 % FBS (10099–141, Gibco) + 100 U/mL penicillin (15070–063, Gibco) + 100 μg/mL streptomycin (11860–031, Gibco) for 24 h. Non-adherent cells were harvested and incubated on the dishes containing M-CSF (40 ng/mL; HZ-1192, Proteintech, IL, USA) for 3 days. The adherent cells were used as OCPs and induced with M-CSF (30 ng/mL) and RANKL (100 ng/mL; Eg1956, Proteintech) for 4 days in alfa-MEM supplemented with 10 % FBS and 1 % penicillin-streptomycin, as previously described [10].

### Viral infection/injection

Lentiviral vectors encoding TF-shRNA were purchased commercially (HANBIO, China). OCPs under good culture conditions were seeded into 6-well plates and cultured at 37 °C in 5 % CO_2_ overnight. Lentiviruses were added into the fresh FBS-free medium at a multiplicity of infection (MOI) of 30 along with 5 μg/mL polybrene. After 24 h, the medium was changed to the fresh FBS-contained medium. Two days later, the infected cells were selected by 7.5 μg/mL puromycin. TF silencing efficiency was evaluated using Western-Blotting assay.

Additionally, model mice were treated with control-shRNA adeno-associated viruses (AAVs) or F7-shRNA AAVs (5 × 10^10^ PFU/mL, marrow-cavity injection; Genechem, Shanghai, China) after exposing the distal femurs via medial patellar displacement surgery and drilling a hole in bone marrow cavity using a 25-gauge needle. After 45 days of intervention, all mice were sacrificed via cervical dislocation under anaesthesia. All animal experimentals were approved by the Ethics Committee of Affiliated Hospital of Putian University (20230076-X701).

### TRAP staining assay

Osteoclast differentiation was evaluated by TRAP staining using a TRAP staining kit (Sigma-Aldrich) following the manufacturer’s instructions. TRAP-positive multinucleated cells (≥3 nuclei) were counted as mature osteoclasts, and TRAP-positive large cells (≥10 nuclei) were counted as large osteoclasts [10].

### Transwell migration assay

Transwell migration assays were performed using 8 μm pore-size Transwell chambers to evaluate the migratory capacity of osteoclast precursors (OCPs). Prior to cell seeding, the Transwell filter membranes were rehydrated with serum-free alfa-MEM at room temperature for 30 min without additional matrix coating. OCPs suspended in serum-free alfa-MEM were seeded into the upper chamber, while alfa-MEM supplemented with 20 % fetal bovine serum (FBS) and different concentrations of F7 was added to the lower chamber to form a chemotactic gradient. After 24 h of incubation, cells that migrated to the underside of the filter membrane were fixed, stained, and quantified by microscopic counting.

### Western-Blotting assay

Total protein was extracted from OCPs. Protein samples were separated by SDS-PAGE, transferred to PVDF membranes, and probed with primary antibody against TF (Abcam, ab239521, 1:2000). Secondary horseradish peroxidase-conjugated antibody was used, and signals were detected by ECL chemiluminescence (ChemiDoc, Bio-Rad; CA, USA).

### Quantitative real-time PCR (qPCR) assay

Total RNA was extracted using TRIzol reagent. cDNA was synthesized, and qPCR was performed with specific primers for TRAP, MMP9, CTSK, and GAPDH using a SYBR Green PCR Kit. Relative gene expression was calculated by the 2^−^ΔΔCt method [11]. The sequences of designed primers are following: *TRAP-*Forward (5′–3′): GCT​GGA​AAC​CAT​GAT​CAC​CT; *TRAP-*Reverse (5′–3′): TTG​AGC​CAG​GAC​AGC​TGA​GT; *MMP9-*Forward (5′–3′): CCT​GTG​TGT​TCC​CGT​TCA​TCT; *MMP9-*Reverse (5′-3′): ACC​CGA​ATC​TAG​TAA​GGT​CGC; *CTSK-*Forward (5′–3′): GGA​AGA​AGA​CTC​ACC​AGA​AGC; *CTSK-*Reverse (5′–3′): GTC​ATA​TAG​CCG​CCT​CCA​CAG; *GAPDH-*Forward (5′–3′): ACC​ACA​GTC​CAT​GCC​ATC​AC; *GAPDH-*Reverse (5′–3′): TCC​ACC​ACC​CTG​TTG​CTG​TA.

### Bone tissue isolation

Mouse femurs and tibias were harvested, cleaned of soft tissues, and either fixed in 4 % paraformaldehyde for subsequent staining or stored at −80 °C for other analyses.

### Micro-CT scanning

Isolated femurs were scanned using a Micro-CT system (Bruker Skyscan 1276; Kontich, Belgium) and observed area is located in the cancellous bones in proximal tibial metaphysis. The scanning settings were as follows: voxel size 7.03 μm, medium resolution, 55 kV, 200 mA, 1 mm Al filter and integration time 387 ms. Trabecular bone parameters including Bone Mineral Density (BMD), Bone Volume/Total Volume (BV/TV), Trabecular Number (Tb.N), Trabecular Thickness (Tb.Th), Trabecular Separation (Tb.Sp) and Bone Surface/Bone Volume (BS/BV) were analyzed with dedicated software.

### Histological and immunofluorescence staining of bone tissues

Fixed bone tissues were decalcified, embedded in paraffin, and sectioned. H&E staining and TRAP staining were performed to evaluate trabecular structure/areas and osteoclast number, respectively. For immunofluorescence staining, the sections were incubated with primary antibody against RANK (Santa Cruz Biotechnology, sc-374360, 1:200), followed by fluorescent secondary antibody, and observed under a fluorescent microscope (Leica DMI8; Wetzlar, Germany).

### ELISA assay of mouse serum

Mouse serum was collected by orbital bleeding. Serum levels of C-terminal telopeptide of type I collagen (CTX) and Procollagen type I N-terminal propeptide (PINP) were measured using commercial ELISA kits (R&D Systems) according to the manufacturer’s protocols.

### Statistical analysis

All experiments were repeated at least three times. Data were presented as mean ± standard deviation (SD). Statistical differences were analyzed using GraphPad Prism 8.0 software. Student’s t-test or one-way ANOVA was used, and p<0.05 was considered statistically significant.

## Results

### F7 promotes OCP migration

Firstly, *in vitro* Transwell migration assays showed that adding F7 to lower lumen of Transwell significantly promoted the migration ability of OCPs in a concentration-dependent manner, and the pro-migration ability of 10 nM of F7 was the strongest (Figure 1A and B). The contribution of F7 to OCP migration is accordingly demonstrated.

**Figure 1:**
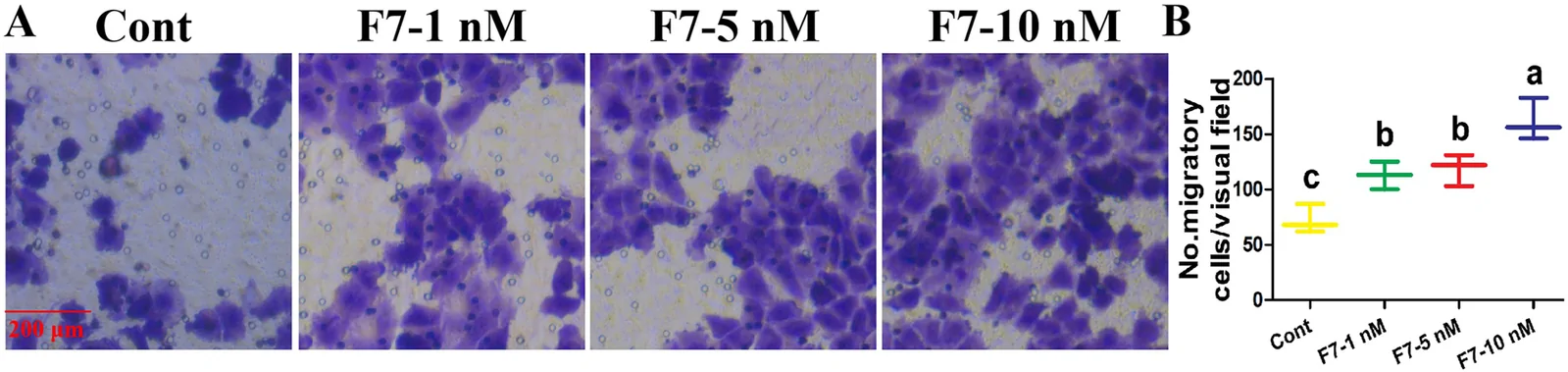
F7 promotes OCP migration and osteoclast differentiation. (A, B) OCP migration after treatment with different levels of F7 for 24 h. Scale bar, 200 μm. The demotion in letters (such as a-b, b-c and c-d) indicates a significant decrease with p<0.05 by one-way ANOVA and Tukey post-Hoc multiple comparisons. Cont, control group.

### F7 promotes osteoclast differentiation

TRAP staining results also showed that treatment with F7 significantly enhanced the differentiation from OCPs towards osteoclasts, manifested as an increase in the number and size (TRAP-positive large cells (≥10 nuclei) are used to evaluate cell size) of mature osteoclasts in a concentration-dependent manner, and the pro-differentiation ability of 10 nM of F7 was the strongest (Figure 2A–D). The expression levels of osteoclastic genes (TRAP, MMP9 and CTSK) presented by qPCR assays also increased in a concentration-dependent manner upon treatment with F7, consistent with TRAP staining results (Figure 2E–G). Consistently, 10 nM of F7 exhibited the strongest gene expression (Figure 2E–G). The contribution of F7 to osteoclast differentiation is also demonstrated.

**Figure 2:**
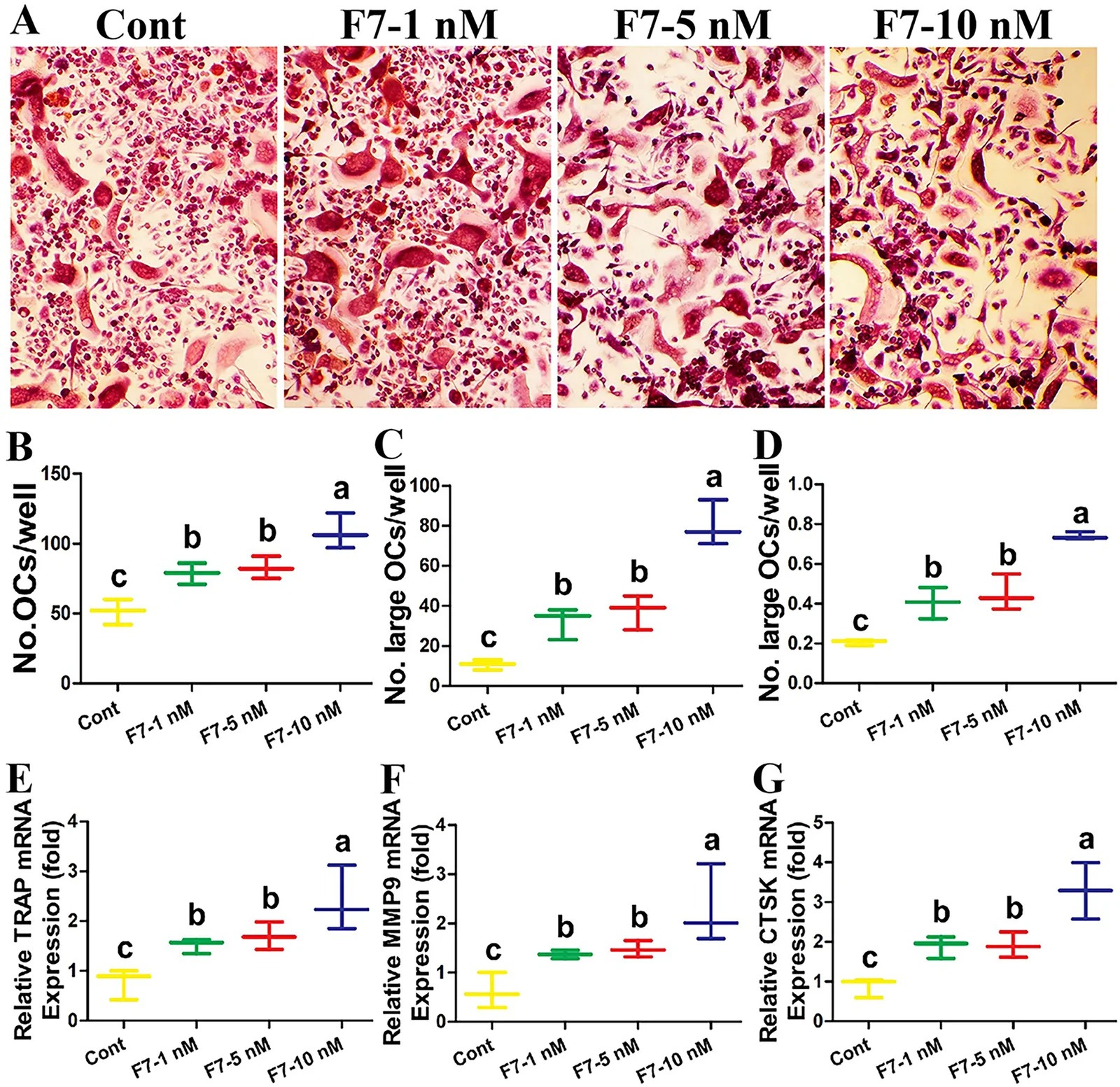
F7 promotes osteoclast differentiation. (A–D) osteoclast differentiation after treatment with different levels of F7 (pretreatment for 30 min) for 4 days under osteoclastic induction. Scale bar, 200 μm. TRAP-positive multinucleated cells (≥3 nuclei) were counted as mature osteoclasts; TRAP-positive large cells (≥10 nuclei) were counted as large osteoclasts and the quantitative determination of large osteoclasts is achieved through cell counting and their proportion analysis. (E–G) mRNA levels of *TRAP*, *MMP9* and *CTSK* in cells after treatment with different levels of F7 for 4 days under osteoclastic induction. The demotion in letters (such as a-b, b-c and c-d) indicates a significant decrease with p<0.05 by one-way ANOVA and Tukey post-Hoc multiple comparisons. Cont, control group.

### F7-promoted OCP migration are blocked by TF silencing

In F7-TF complex, the function of F7 needs to be implemented via TF. We also need to clarify the relationship between F7 and TF in the OCPs-regulating roles. TF silencing efficiency was verified by gene and protein detection (Figure 3A and B). Transwell migration assays showed that TF silencing blocked OCP migration enhanced by F7 treatment, and there was no significant difference in the migration ability of TF silencing groups regardless of whether F7 was added or not (Figure 3C and D). Accordingly, the significance of TF for F7-regulated OCP migration is elucidated.

**Figure 3:**
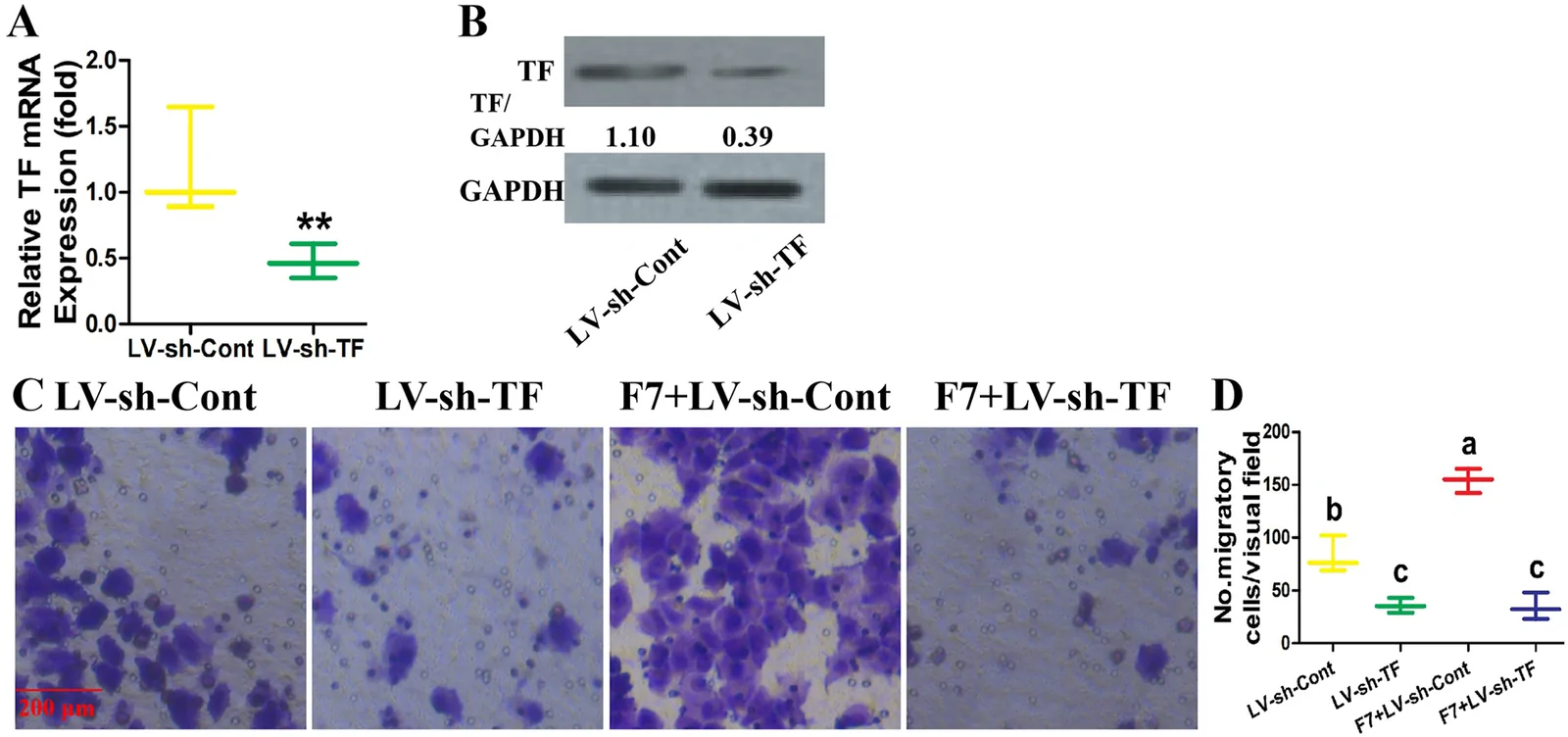
F7-promoted OCP migration are blocked by TF silencing. (A, B) mRNA and protein detection to demonstrate TF silencing efficiency and (C, D) OCP migration after treatment with F7 and/or TF-shRNA for 24 h. Scale bar, 200 μm. The demotion in letters (such as *a*-*b* and *b*-*c*) indicates a significant decrease with p<0.05 by one-way ANOVA and Tukey post-Hoc multiple comparisons. LV-sh-Cont, control-shRNA lentiviruses; LV-sh-TF, TF-shRNA lentiviruses.

### F7-promoted osteoclast differentiation are blocked by TF silencing

TRAP stainings also showed that TF silencing blocked the number and size of mature osteoclasts promoted by F7 treatment, and there was no significant difference in the osteoclast differentiation of TF silencing groups regardless of whether F7 was added or not (Figure 4A–D). The increases in the expression levels of osteoclastic genes (TRAP, MMP9 and CTSK) as F7 addition were blocked by TF silencing (Figure 4E–G). The significance of TF for F7-regulated osteoclast differentiation is also elucidated.

**Figure 4:**
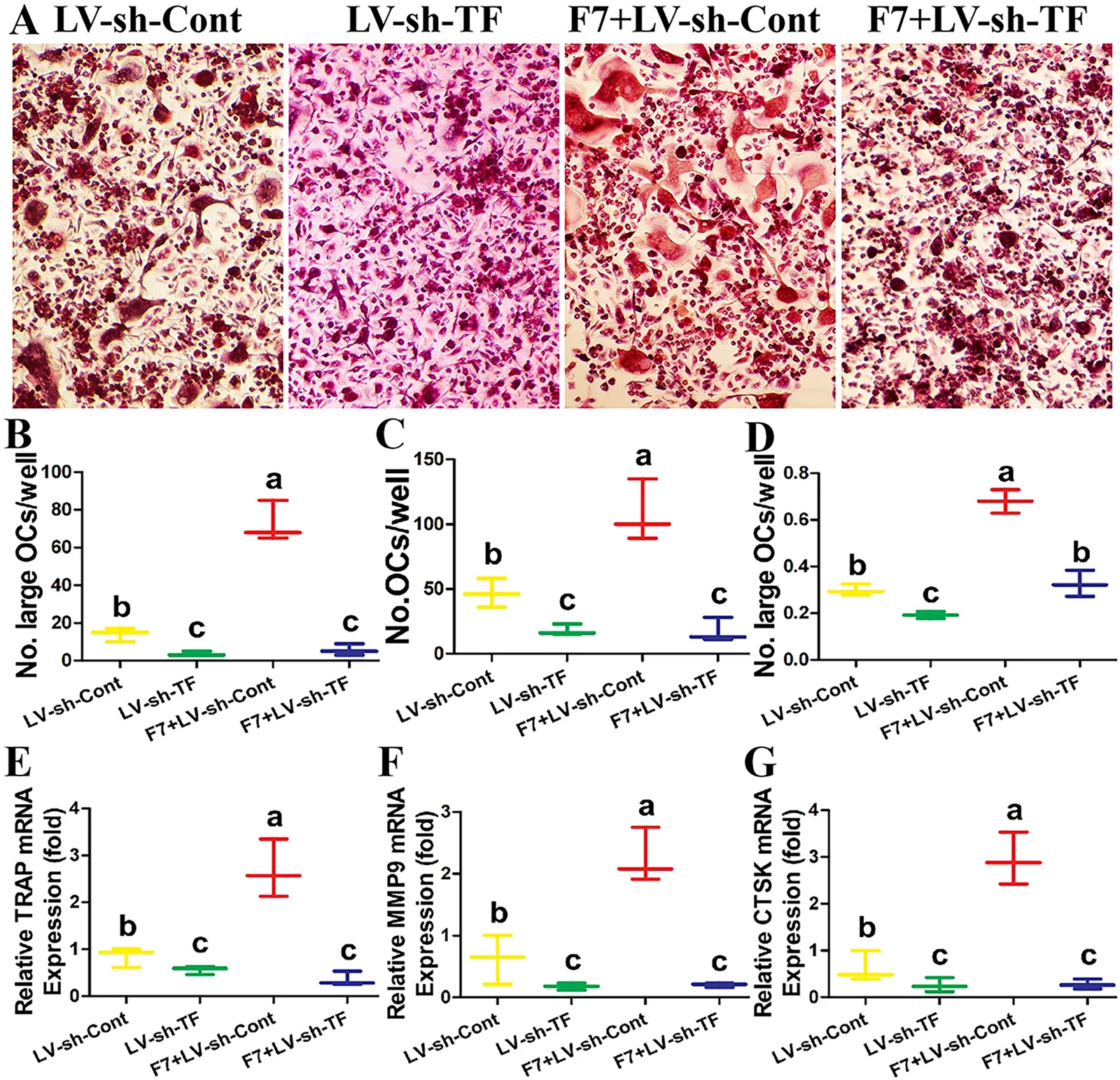
F7-promoted osteoclast differentiation are blocked by TF silencing. (A–D) osteoclast differentiation after treatment with F7 (pretreatment for 30 min) and/or TF-shRNA for 4 days under osteoclastic induction. Scale bar, 200 μm. TRAP-positive multinucleated cells (≥3 nuclei) were counted as mature osteoclasts; TRAP-positive large cells (≥10 nuclei) were counted as large osteoclasts and the quantitative determination of large osteoclasts is achieved through cell counting and their proportion analysis. (E–G) mRNA levels of *TRAP*, *MMP9* and *CTSK* in cells after treatment with F7 and/or TF-shRNA for 4 days under osteoclastic induction. The demotion in letters (such as *a*-*b* and *b*-*c*) indicates a significant decrease with p<0.05 by one-way ANOVA and Tukey post-Hoc multiple comparisons. LV-sh-Cont, control-shRNA lentiviruses; LV-sh-TF, TF-shRNA lentiviruses.

### F7 silencing improves bone loss in OVX mice *in vivo*

*In vivo* micro-CT assays showed that F7 silencing effectively improved the phenotype of trabecular bone loss in OVX mice (Figure 5A). Corresponding quantitative parameters showed that decreased BMD, BV/TV, Tb.N, and Tb.Th as well as increased Tb. Sp and BS/BV in OVX mice were recovered by F7 silencing (Figure 5D). H&E staining results showed that the inadequate trabecular area in OVX mice was improved by F7 silencing (Figure 5B–E). TRAP staining results showed that the increase in the osteoclast number in trabecular bones of OVX mice was reversed by F7 silencing (Figure 5C–F). Additionally, ELISA assays showed that increased production of serum CTX cytokines from OVX mice was reversed by F7 silencing (Figure 5G). The role of F7 in osteoporotic phenotype is accordingly confirmed.

**Figure 5:**
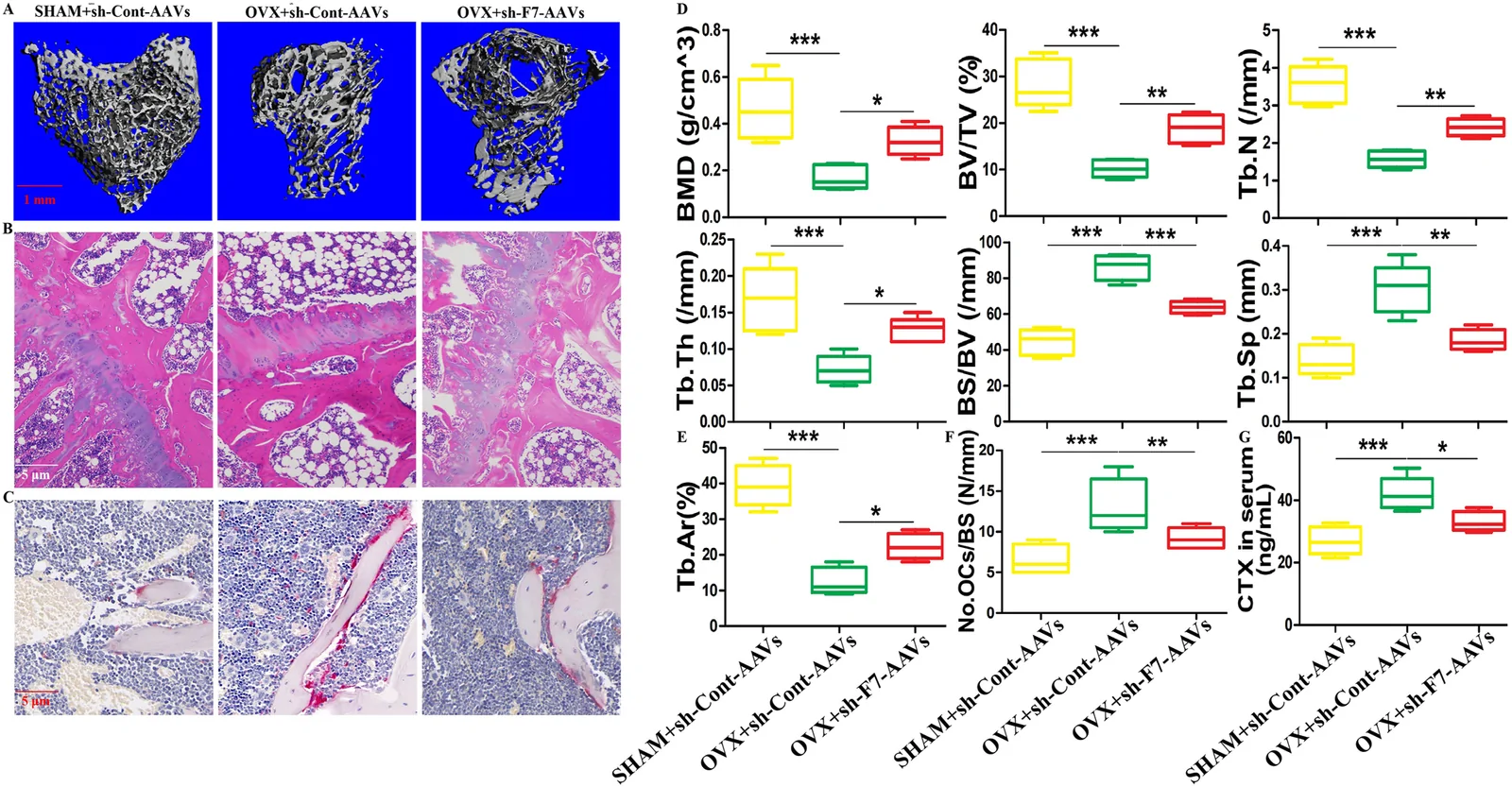
F7 improves bone loss in OVX mice *in vivo*. Mice were sham operated or ovariectomized, and OVX mice were treated with F7-shRNA-AAVs for 45 days. (A) Representative 3D Micro-CT reconstructed images of tibiae from each group. Scale bar, 1 mm, (B) representative H&E-stained femoral sections from each group. Scale bar, 5 μm, (C) representative TRAP-stained femoral sections from each group. Scale bar, 5 μm, (D) Micro-CT analyses showing trabecular bone parameters, including BMD, BV/TV, Tb.N, Tb.Th, BS/BV and Tb. Sp (n=5), (E) H&E analyses showing trabecular bone area (%Tb.Ar) (n=5), (F) TRAP analyses showing osteoclast number on the surface of trabecular bones (n=5) and (G) CTX serum levels were detected by using ELISA kits (n=5). *p<0.05, **p<0.01, ***p<0.001 by one-way ANOVA and Tukey post-Hoc multiple comparisons. sh-Cont-AAVs, control-shRNA AAVs; sh-F7-AAVs, F7-shRNA AAVs.

### F7 silencing reduces trabecular RANK^+^ cells in OVX mice *in vivo*

Given that RANK in trabecular bones is a marker for OCPs and mature osteoclasts, the detection of RANK^+^ cells in trabecular bones can be used to evaluate the migration of OCPs towards bone matrix. Notably, the increase in the number of RANK^+^ cells in trabecular bones of OVX mice was reversed by F7 silencing (Figure 6A and B), indicating the *in vivo* role of F7 in OCP migration. The role of F7 in OCP-bone matrix adhesion is suggested.

**Figure 6:**
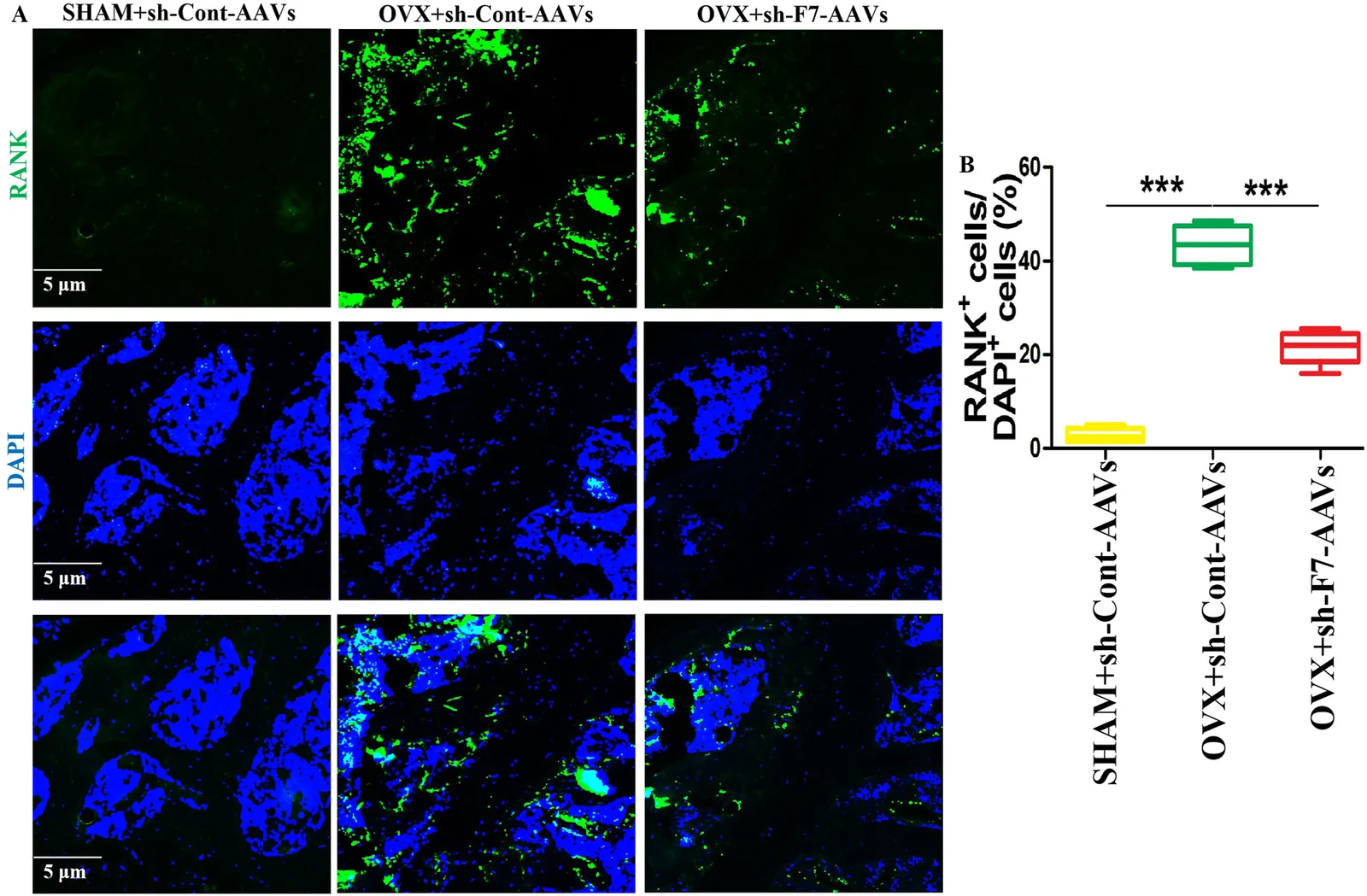
F7 silencing reduces trabecular RANK^+^ cells in OVX mice *in vivo*. Mice were sham operated or ovariectomized, and OVX mice were treated with F7-shRNA-AAVs for 45 days. (A) Representative RANK-fluorescence-stained femoral sections from each group. Scale bar, 5 μm and (B) RANK-fluorescence analyses showing the number of RANK^+^ cells on the surface of trabecular bones (n=5). ***p<0.001 by one-way ANOVA and Tukey post-Hoc multiple comparisons. sh-Cont-AAVs, control-shRNA AAVs; sh-F7-AAVs, F7-shRNA AAVs.

## Discussion

In the present study, we found that F7 is a key regulatory factor promoting osteoclastogenesis. Given that the corresponding receptor, TF, is markedly expressed in monocytes/macrophages [12], [13], [14], the role of F7 in OCP migration through its pro-migratory effect has great research value. Notably, OCP migration is a critical prerequisite for OCP recruitment to form mature osteoclasts and reach bone resorption sites [6], 7]. Current data reveal that F7 enhances OCP migration by binding to TF, thereby facilitating osteoclast recruitment and differentiation, and ultimately promoting bone loss in OVX mice. A working model of our findings is illuminated in Figure 7. Osteoclast differentiation is known to be tightly regulated by M-CSF and RANKL signaling, which mediate global epigenetic and transcriptional changes [15], 16]. Our findings supplement this regulatory network by identifying F7 as a novel participant in osteoclastogenesis. This finding suggests that targeting F7 may provide a novel potential strategy for the treatment of osteoclastic osteoporosis (e.g., postmenopausal osteoporosis).

**Figure 7:**
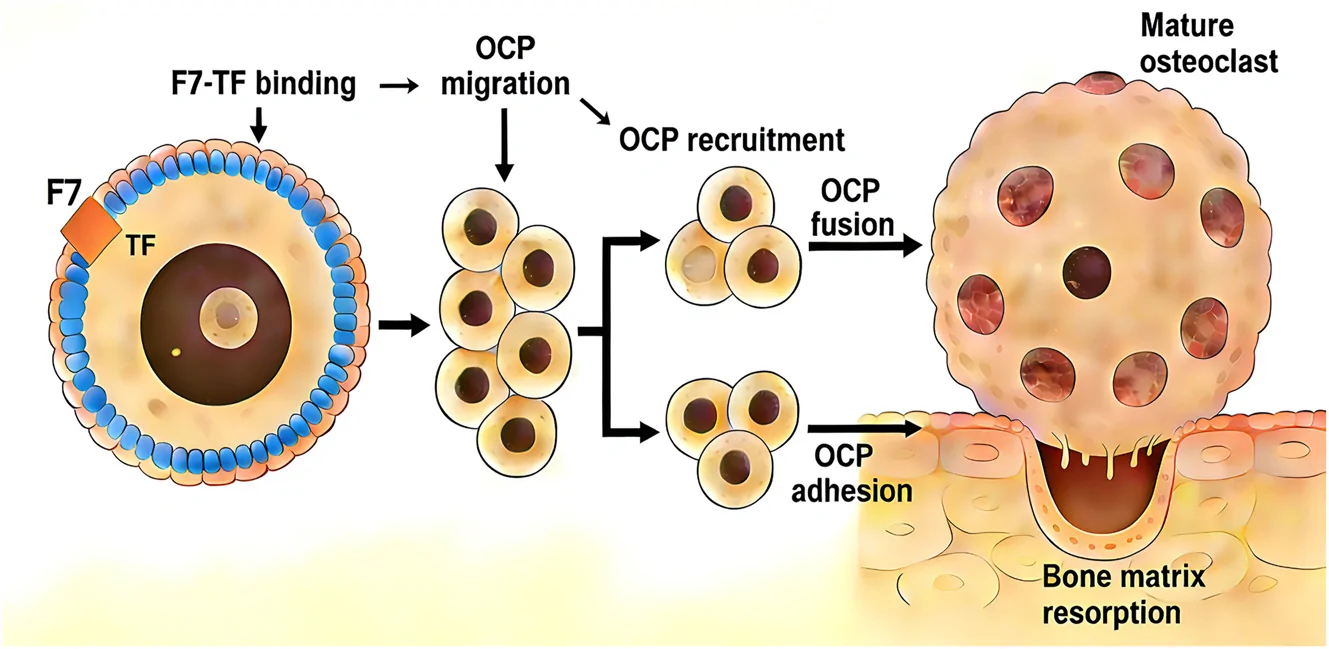
Working model of F7’s mechanism of action. F7 promotes OCP migration and recruitment by binding to TF, leading to OCP fusion and osteoclast differentiation and maturation, as well as the adhesion of OCPs to bone matrix sites for bone resorption.

However, the physiological function of F7 must be emphasized. As a core coagulation factor, inhibition of F7 activity may lead to coagulopathy and increase the risk of bleeding [17], 18]. Therefore, future clinical translation needs to address the issue of targeting specificity. At present, small-molecule targeted inhibitors and nanoparticle-based delivery systems have become mature and widely adopted strategies for the development of therapeutic agents against osteoclast-related bone diseases. Emerging evidence supports antibody-mediated targeted delivery as another promising strategy; for example, anti-RANK antibody-drug conjugates have been successfully developed to selectively target osteoclasts and inhibit bone resorption without obvious systemic side effects [19]. Following this rationale, designing OCP-specific antibody-drug conjugates that accurately localize to the F7-TF complex on the OCP surface could avoid interference with the systemic coagulation system. Meanwhile, regular monitoring of coagulation function and routine blood parameters during treatment is necessary to guarantee therapeutic safety.

Subsequent studies can further characterize the downstream signaling pathways through which F7-TF signaling governs OCP migration. Multiple key regulators of osteoclastogenesis, including the transcription factor KLF7 and the m^6^A demethylase FTO, have been validated to modulate OCP differentiation, proliferation, and survival [20], 21]. Future work should investigate whether F7-TF signaling crosstalks with these established regulators or triggers specific signaling cascades to control OCP migration and osteoclast formation. Elucidating these mechanisms will help identify more precise therapeutic targets for drug development and facilitate the clinical translation of F7-targeted strategies for treating osteoporosis driven by excessive osteoclastic bone resorption.

## Conclusions

In summary, this study establishes F7 as a pivotal regulator for OCP migration, a previously underappreciated but crucial step during osteoclastogenesis, thereby expanding the mechanistic landscape of bone resorption control. Once the safety issues associated with F7 targeting are resolved, our research will provide further insights into improving osteoporosis treatment.
